# The Pendulum Swings Both Ways: Evidence for U-Shaped Association between Sleep Duration and Mental Health Outcomes

**DOI:** 10.3390/ijerph20095650

**Published:** 2023-04-26

**Authors:** Karolina Kósa, Szilvia Vincze, Ilona Veres-Balajti, Éva Bácsné Bába

**Affiliations:** 1 Department of Behavioural Sciences, Faculty of Medicine, University of Debrecen, 4032 Debrecen, Hungary; 2 Department of Sectoral Economics and Methodology, Faculty of Economics and Business, University of Debrecen, 4032 Debrecen, Hungary; 3 Department of Physiotherapy, Faculty of Health Sciences, University of Debrecen, 4032 Debrecen, Hungary; 4 Department of Sports Economy and Management, Faculty of Economics and Business, University of Debrecen, 4032 Debrecen, Hungary

**Keywords:** sleep duration, psychological stress, sense of coherence, life satisfaction, work ability

## Abstract

Short sleep duration is a known risk to health, but less certain is the impact of longer sleep duration on various measures of health. We investigated the relationship between sleep duration and mental health outcomes in a cross-sectional survey conducted on a homogenous sample of healthy governmental employees (N = 1212). Data on sleep duration, subjective health, psychological stress, sense of coherence, life satisfaction and work ability along with sociodemographic data were collected. Sleep duration was significantly longer, and mental health outcomes and work ability were significantly better among those in at least good subjective health. Fitting mental health outcomes on sleep duration suggested a quadratic or fractional polynomial function, therefore these were tested and the best-fitting models were selected. Longer than 8 h of sleep duration was associated with a decreasing sense of coherence and decreasing work ability. However, psychological stress and life satisfaction were positively impacted by more than 8 h of sleep. Sleep duration likely has an optimum range for health, similar to other variables reflecting homeostatic functions. However, this is difficult to prove due to the left-skewed distribution of sleep duration.

## 1. Introduction

Ever since its inception in the 1970s [1], the field of sleep medicine has provided ample evidence for the importance of sleep for maintaining homeostasis [2] and reducing the risk of a wide variety of diseases and mortality [3,4]. Considerable research has been dedicated to diagnosing and treating patients suffering from sleep disorders such as insomnia, sleep apnea and restless leg syndrome [5,6]. In addition to medical conditions impairing sleep, another large-scale problem has been identified in recent decades, namely, sleep deprivation caused by a host of potentially amenable non-medical factors. The first national report from the USA on sleep disorders estimated, in 1993, that total sleep time had decreased by 20% in the country over the past century [7]. Less than 7 h per night is considered short sleep duration [8], and, according to recent data, more than a third of adults in the USA are short sleepers [9]. Studies of sleep epidemiology [10] have revealed that sleep deprivation has become a global problem, increasing not only the risk of a wide range of diseases but impairing cognitive and work performance and mental health, as well [11].

However, long sleep duration also entails health risks, as was first revealed by the analysis of follow-up data from the 1982 Cancer Prevention Study II of the American Cancer Society, which showed that sleep duration above 8 h linearly increased mortality hazard [12]. Subsequent meta-analyses also found evidence for an association between both shorter (<7 h) and longer (>8 h) sleep duration and premature all-cause mortality [13,14]. A recent large-scale systematic review also found evidence for long compared to normal sleep duration being significantly associated with incident diabetes and cardiovascular diseases [15]. Brazilian researchers found that self-reported long (>8 h) and short (<7 h) sleep duration both were associated with shorter telomere length, an indicator of age-related diseases [16]. In light of these findings, a U-shaped association between sleep duration and health outcomes was proposed. Our aim was to investigate whether a U-shaped association can be detected between sleep duration and mental health variables in a relatively homogenous sample of nonmanual workers with tertiary education.

## 2. Materials and Methods

### 2.1. Study Design and Sample

The cross-sectional study was carried out in two large governmental institutions by inviting all employees to fill in an online questionnaire approved by the appropriate ethics body and the leadership of both institutions. All 6927 employees were invited by email to participate in the autumn of 2015. Participation was voluntary and anonymous; no personal data was collected.

### 2.2. Study Variables

The sociodemographic variables included birth year, from which age was computed for 2015, gender and marital status (single, married/cohabiting, separated/divorced or widowed). Marital status was collapsed into a binary variable with two categories: one for those who live with a partner (married or cohabiting), and another for those who live alone (single, separated, divorced or widowed). Educational status was reported as primary, secondary or tertiary education. Since there was no respondent with primary education in the sample, education was, in fact, a binary variable.

Sleep duration was reported as the average rounded natural number of hours of sleep on workdays of the past week in an open-ended fashion. Extremely low duration (less than 3 h, three records) was allocated to 3 h of sleep; extremely high duration (more than 9 h; two records) was allocated to 10 h of sleep for fitting and modeling. The number of hours of sleep was collapsed into three categories for descriptive statistical analysis, defining the categories of sleep duration based on the literature [2].

The health outcomes were measured by validated scales. The perceived or subjective health was measured by the Hungarian version of the standard item recommended by the World Health Organization [17], answerable on a five-point Likert scale. The responses were collapsed into two categories for analysis: at least good (if very good and good) and less than good (if acceptable, bad, or very bad). Psychological distress was measured by the Hungarian version [18] of the 12-item General Health Questionnaire (GHQ-12) [19]. There are several options to evaluate the GHQ-12, of which the total score was used in the present study. The sense of coherence, a dynamic sense of confidence with a global orientation to view life as structured, manageable and meaningful [20], was measured by the Hungarian version [21] of the 13-item Sense of Coherence scale [22]. The item for life satisfaction was taken from the European Health Interview Survey assessed on a Cantril Scale (1: not satisfied at all, 10: fully satisfied) [23]. Current work ability was measured by one item on a Cantril Scale (0: not able to work, 10: best work ability), taken from the Hungarian version of the Work Ability Index [24].

### 2.3. Confounders

One item on employment status inquired about being in a managerial position or not (yes/no) since all the respondents were employees of the respective institutions. Health behavior was assessed by one item on smoking (current smoker/former smoker/never smoker). Social support was measured by the Hungarian version of the respective scale of the Health and Lifestyle Survey and Health Survey for England [25]. Briefly, seven items were answered, each on a 1–3 scale. The overall scores ranged between 7 and 21, with the maximum score of 21 indicating no lack of social support. Scores of 18 to 20 indicated a moderate lack of social support, whereas scores of 17 or less reflected a severe perceived lack of social support.

The items assessing sociodemographic variables, life satisfaction and smoking were taken from the Hungarian version of the questionnaire of the European Health Interview Survey of 2014 [26].

### 2.4. Statistical Analysis

Chi-square tests were used to examine the relationship between the categorical variables. For describing the sample, sleep duration was converted into a categorical variable with 3 categories (less than 7 h, 7 h and more than 7 h of sleep) since seven hours is the lowest sleep duration supporting optimal health in adults [8], which was also characteristic for the largest number of respondents.

Various fit functions, such as linear prediction, quadratic prediction and local polynomial smoothing, were used to create graphical representations of the association between sleep duration and measures of health, all defined as continuous variables. The choice of fit functions was based on papers suggesting a U-shaped association between sleep duration and health outcomes [13,14], pointing to a quadratic or a polynomial function between predictors and outcomes. Since heteroskedasticity was detected for sleep duration and mental health outcomes, robust estimations for the standard error of coefficients are reported. All the statistical analyses were performed by Stata/IC 16.1 for Windows (2019, Statacorp LLC, Lakeway Drive, TX, USA).

## 3. Results

### 3.1. Description of the Sample

Altogether, 1212 people filled in the online questionnaire (32.01% male, 67.99% female) of whom 14.46% were below 30 years of age, 79.01% were between 30–59 years, and 6.52% were 60 years or above. Of these, 62.35% lived with a partner. The vast majority had tertiary education (86.54%), 13.46% had secondary education, and none of the respondents had only primary education. A total of 12.65% of the respondents had managerial positions in their institution; the rest (87.35%) had non-managerial positions. Two-thirds of the respondents never smoked (66.98%), 14.98% were current smokers, and the rest were former smokers (18.04%). More than two-thirds (69.27%) had optimal social support, and only 11.47% perceived a severe lack of support. The study group was quite homogenous in terms of mostly comprising young–middle-aged women in non-managerial positions with tertiary education.

Since the distribution of sleep duration was left-skewed, the mean of sleep duration (6.83 ± 0.98 h) was below the median (7 h, IQR: 6; 7.5). The skewness of all the variables that were considered continuous in the modeling (sleep duration, age, psychological stress, sense of coherence, life satisfaction, work ability) was below 2.0, indicating that they can be treated as normally distributed [27].

Considering sleep duration as an interval variable, significant differences in sleep duration were found by gender (females sleeping 0.12 h more, *p* = 0.031), age groups (*p* = 0.046), employment position (*p* = 0.003), and social support (*p* < 0.001). However, sleep duration did not significantly differ by gender and age groups when the categories of sleep duration were examined, as shown in Table 1, which describes the characteristics of the study population by sleep duration as a categorical variable with three categories. As shown in Table 1, the categories of sleep duration were significantly associated with employment position (with a higher proportion of managers having less than 7 h of sleep) and social support, but not with gender and age group.

### 3.2. Sleep Duration and Mental Health by Subjective Health

Demographic and mental health variables are described by subjective health, which was collapsed into two categories: less than good and at least good, as described in Methods. Those who perceived their health as good or very good were significantly younger, more of them had tertiary education, had optimal social support, and had significantly longer sleep duration. In addition, all the mental health indicators were significantly more favorable in this group compared to those who perceived their health as less than good: a significantly lower proportion of them were highly stressed, they scored more than 5 points higher in terms of sense of coherence, had a 0.83 points higher score for satisfaction with life, and 0.6 points higher score for self-perceived work ability (Table 2).

### 3.3. Effect of Sleep Duration on Measures of Mental Health

The effect of sleep duration on mental health outcomes (psychological distress, sense of coherence, life satisfaction and work ability) was first investigated graphically by fitting linear, quadratic and polynomial functions, as described in Methods.

**Figure 1:**
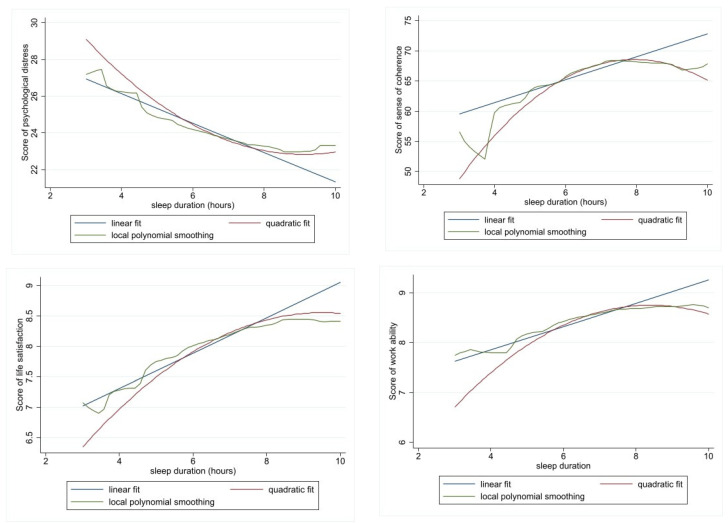
Various fits for the function of sleep duration on four indicators of mental health.

The quadratic fit and polynomial smoothing produced downward-sloping and U-shaped curves for psychological stress as a negative indicator of mental health, and upward-sloping and inverse U-shaped curves for the other three positive indicators of mental health (Figure 1).

The non-linear curves were noticeably different from the linear fit. Therefore, we proceeded to test different models for the effect of sleep duration on mental health variables. First, we carried out a Spearman rank-order correlation to decide which covariates should be included in the modeling. This nonparametric version of the Pearson correlation was used in order to include factor variables, as well, and check their association with sleep duration and mental health measures. Spearman correlation assumes a monotonic relationship between two variables, which was not held for our independent and outcome variables, but the major point was to decide which potential confounders to include in further modeling. The results are shown in Table 3, revealing that smoking had no correlation with any other variables, so it was not included in the models. Subjective health, as a categorical health outcome, was also not included in the models to reduce collinearity, since subjective health showed a significant correlation with all mental health outcomes.

Next, we tested the effect of sleep duration on mental health outcomes by performing multivariate linear regression, quadratic and fractional polynomial regression for each mental health outcome separately in order to decide which model would best predict the outcomes. The selected confounders were included in all the models. Sleep duration was considered a normally distributed predictor, ranging from 3 h to 10 h. Outliers of sleep duration were removed by collapsing them into the lowest and highest number of sleep duration as described in Methods. All four mental health variables were standardized before modeling to facilitate a comparison of the model parameters. Mental health outcome variables, as well as sleep duration and age as independent variables, were used as continuous ones in the models; all the other covariates selected by correlation were binary. Table 4 gives the summary of the models, showing only p values for the effect of sleep on the outcome variables and the goodness-of-fit parameters of the model.

Sleep duration had a strong significant positive impact on all mental health outcomes. Selection of the best fitting model for predicting the effect of sleep duration on mental health outcomes was made after taking into account the alpha value of sleep duration in the model, the model parameters, such as the highest adjusted regression coefficient (R2), lowest root mean square error (RMSE), and lowest Akaike’s information criterion (AIC), as well as regression diagnostics including residuals, multicollinearity, omitted variables and data points with high leverage. Fit and residual plots were also inspected (not shown). The best model was the fractional polynomial for psychological distress and sense of coherence, and the quadratic for life satisfaction and work ability (Table 4).

Of all the models, age—instead of sleep duration—proved to be the fractional polynomial (age = [age/10]^3^—70.38) for psychological stress. Sleep duration for sense of coherence was defined by two polynomials (X1[hours]^2^—46.59), and (X2[hours]^3^—318), respectively. Life satisfaction was equally well predicted by all three models according to the fit statistics, but there remained many leverage points after the linear, few after the polynomial and none after the quadratic regression. All the outcomes were acceptably modeled by sleep duration and the covariates, as shown by the values of the coefficients of determination, except work ability. This was also seen by post-estimation tests for the omitted variables (for psychological stress: *p* = 0.322, for sense of coherence: *p* = 0.061, for life satisfaction: *p* = 0.127 and for work ability: *p* = 0.001).

### 3.4. Estimating Parameters in the Best Models

After identification of the best model for each outcome, the selected regression was run to estimate each mental health variable, including the preselected demographic and other covariates (gender, age, living with a partner, education, employment position and social support). Because heteroscedasticity was identified in terms of sleep duration and all the mental health outcomes, robust estimates are shown in Table 5.

Sleep duration had a significant positive impact on all mental health outcomes. For psychological stress, negative scores for most independent variables were expected since lower scores reflect lower levels of stress. Therefore, a decrease in the standard deviation from the mean by increasing sleep duration reflects improvement.

In addition to sleep duration, two other covariates had strongly significant positive effects on all mental health outcomes: optimal social support (vs less than optimal support) and employment position, namely being in a leadership position (manager) compared to being nonmanager. (Both being a manager and having optimal social support decrease the distance from the means of psychological stress, reflecting improvement).

The effects of other covariates varied by health outcome. Being female was a positive predictor for life satisfaction and work ability, while age was a positive predictor for psychological stress only (decreasing deviation from the mean). Living with a partner and having tertiary education were significant predictors for sense of coherence and life satisfaction.

After estimating predictors, the effect of sleep duration was visualized on each mental health outcome based on the best models shown in Table 4.

**Figure 2:**
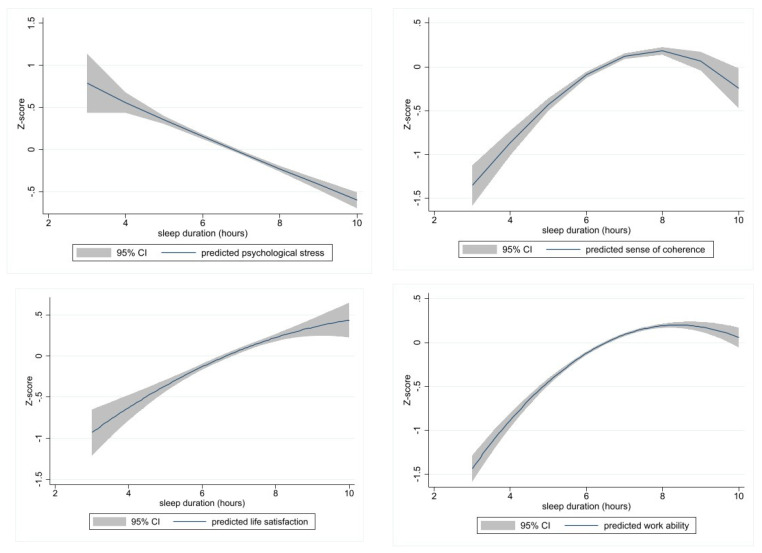
Predictions of the effect of sleep duration on four mental health outcomes according to the best models.

Longer sleep duration improves sense of coherence (Figure 2. upper right panel) and work ability (Figure 2. lower right panel) up to 8 h, but, after that, the effect turns downward (negative). Up to 10 h, increasing sleep duration decreases psychological stress (Figure 2. upper left panel) and increases life satisfaction (Figure 2. lower left panel) in a close-to-linear fashion.

## 4. Discussion

Our study found evidence for a U-shaped association between sleep duration and certain mental health outcomes in a homogenous sample of healthy workers. Longer than 8 h of sleep duration was associated with decreasing sense of coherence and decreasing work ability. This association was not found for psychological stress and life satisfaction, both of which were positively and linearly impacted by more than 8 h of sleep. The association between sleep duration and mental health was found to be nonlinear for all mental health outcomes. The predicted association was in contrast with the best identified model only in the case of psychological stress, where the best model was found to be the fractional polynomial while the prediction suggested a linear association. The models for the linear and quadratic functions between sleep duration and life satisfaction were almost identical; the latter was chosen because of its better post-regression diagnostics. The polynomial model for sense of coherence and the quadratic model for work ability fit well with the prediction regarding the impact of sleep duration on these two outcomes.

Our cross-sectional study, reporting a U-shaped association between sleep duration, sense of coherence, life satisfaction and work ability, cannot provide causal evidence but is suggestive of the existence of an optimal range of sleep duration for mental health. An advantage of the study is the quite homogenous sample with active, healthy, employed persons working in very similar environments. However, the dominance of females and those with tertiary education certainly reflects selection bias in the sample. Subjective recall of sleep duration is less reliable than actigraphy records, but it is unlikely that this would have introduced systematic bias.

A low response rate is a limitation of the study, but it is comparable to other workplace online surveys with no incentive. A 33% online response rate was achieved in a much younger workplace sample in a Finnish study [28]. A recent study of Chinese workers returning to work from home during the pandemic found that only 9.4% (N = 163) of all contacted enterprises with turnovers of at least 20 million RMB approved participation, and, of these, altogether 2410 workers completed voluntarily an online questionnaire (14.8 respondents per enterprise) [29]. A low response rate may lead to an underestimation of prevalence measures but does not seem to impact the strength of association (in our study, between sleep and mental health) [30]. The strength of the relationship between the best models and the dependent variables reflected by the regression coefficient was lowest for work ability, which is likely due to the very limited number of explanatory variables included in the model.

Our finding for psychological distress is in concert with a community-based study in Australia, in which sleep duration was linearly associated with psychological distress [31]. Regarding psychiatric outcomes, short self-reported sleep duration was found to be associated with an increased risk of depression and anxiety disorders in population samples [32,33,34]. This association is indirectly supported by a recent study among Koreans, which found that weekend catch-up sleep of 1–2 h of duration decreased the risk of depression [35]. Some authors surmised a U-shaped association between sleep duration and symptoms of depression [33,34]. The negative effect of short sleep duration on mortality, morbidity and health risk conditions has been widely supported in the literature [4], but much less evidence has been available for the impact of sleep duration exceeding 8–9 h.

There is strong evidence for a U-shaped relationship between sleep duration and mortality (that is, a longer sleep duration increases the risk of death) [13,14,36]. One of the underlying mechanisms might be telomere shortening, which is associated with both self-reported long and short sleep duration [16]. However, there is much less evidence for a U-shaped association between sleep duration and other measures of health and functioning. That is why the American Academy of Sleep Medicine did not include an upper limit for sleep duration in its recommendation statement [8]. However, an excessive quantity of sleep (≥9 h) has been found to co-occur with a range of neurologic and psychiatric disorders [37], and long sleep duration was significantly associated with mortality and incident cardiovascular disease [15].

There has been evidence for a U-shaped association between sleep duration, C-reactive protein and serum uric acid in women [38], and for increased arterial stiffness in a Taiwanese sample among those who slept more than 8 h [39], but this was not confirmed by a similar subsequent study in a larger multi-ethnic sample [40].

## 5. Conclusions

Our findings support the notion that sleep duration most likely has an optimum range for mental health, similar to other variables reflecting homeostatic functions (e.g., body mass index, fluid intake and body temperature). This range likely varies by different mental health measures. However, the impact of a longer duration of sleep on human functioning has been and will remain difficult to prove, regardless of whether sleep duration in the last decades has shortened [7] or not [41]. This is so because the vast majority of healthy adults with jobs and families do not and cannot spend more than one-third of their days sleeping. Therefore, the distribution of sleep duration tends to be left-skewed, making reliable statistical analysis challenging in the range of longer duration.

## Figures and Tables

**Table 1:** Demographic characteristics of the study population by categories of sleep duration.

Variables	Sleep Duration						*p* Value
	<7 h		7 h		>7 h		
	N	%	N	%	N	%	
**Gender**							0.103
Male	141	36.34	154	39.69	93	23.97	
Female	275	33.37	303	36.77	246	29.85	
**Age group**							0.169
18–29 years	49	28.32	62	35.84	62	35.84	
30–45 years	212	35.16	229	37.98	162	26.87	
46–59 years	131	38.3	126	36.84	85	24.85	
60 ≥ years	25	32.05	31	39.74	22	28.21	
**Lives with partner**							0.829
No	151	33.48	171	37.92	129	28.6	
Yes	263	35.21	277	37.08	207	27.71	
**Education**							0.544
Secondary	55	33.74	57	34.97	51	31.29	
Tertiary	362	34.54	400	38.17	286	27.29	
**Employment position**							0.013
Manager	65	43.62	58	38.93	26	17.45	
Non-manager	348	33.82	397	38.58	284	27.6	
**Smoking**							0.593
Current smoker	67	38.07	66	37.5	43	24.43	
Former smoker	80	37.74	78	36.79	54	25.47	
Never smoked	264	33.55	305	38.75	218	27.7	
**Social support**							0.001
Severe lack	61	47.66	49	38.28	18	14.06	
Moderate lack	87	40.47	76	35.35	52	24.19	
Optimal support	246	31.82	305	39.46	222	28.72	

**Table 2:** Demographic and mental health variables by subjective health.

Variables	Subjective Health		*p*
	Less Than Good	At Least Good	
**Gender (%)**			0.178
Male	27.65	72.35	
Female	31.46	68.54	
**Age (years)**	43.72	40.97	<0.001
**Lives with partner (%)**			0.825
No	29.69	70.31	
Yes	30.29	69.71	
**Education (%)**			<0.001
Secondary	44.17	55.83	
Tertiary	28.16	71.84	
**Employment position (%)**			0.638
Manager	28.38	71.62	
Nonmanager	30.27	69.73	
**Social support (%)**			<0.001
Severe lack	51.56	48.44	
Moderate lack	33.64	66.36	
Optimal support	25.03	74.97	
**Sleep duration (hours)**	6.62	6.91	<0.001
**Psychological distress (GHQ-12, %)**			<0.001
Stressed	52.90	47.10	
Not stressed	27.01	72.99	
**Sense of coherence (mean)**	63.10	68.37	<0.001
**Satisfaction with life (mean)**	7.55	8.38	<0.001
**Work ability (mean)**	8.1	8.7	<0.001

**Table 3:** Bivariate correlation for the predictor (sleep duration), the outcome variables (mental health measures) and covariates, and parameters of the predictor and outcome variables.

	1	2	3	4	5	6	7	8	9	10	11	12	13
1 gender	1.000												
2 age		1.000											
3 lives with partner		0.284 *	1.000										
4 education	−0.107			1.000									
5 employment position	0.144 *	−0.317 *	0.155 *	0.128 *	1.000								
6 smoking						1.000							
7 social support							1.000						
8 sleep duration							0.129	1.000					
9 subjective health		−0.120		0.137 *			0.181 *	0.140 *	1.000				
10 psychol.stress		−0.103			−0.112		−0.304 *	−0.186 *	−0.306 *	1.000			
11 sense of coherence		0.190 *	0.200 *		−0.189 *		0.352 *	0.155 *	0.232 *	−0.489 *	1.000		
12 satisfaction w/ llife			0.209 *		−0.130 *		0.421 *	0.183 *	0.282 *	−0.458 *	0.555 *	1.000	
13 work ability	0.101						0.203 *	0.182 *	0.263 *	−0.319 *	0.358 *	0.406 *	1.000
Mean (±SD)	n/a	41.83 (11)	n/a	n/a	n/a	n/a	n/a	6.83 (0.98)	n/a	23.86 (4.04)	66.77 (11.26)	8.13 (1.65)	8.52 (1.23)
Min-max values		223–85						1−12		15–43	23–91	1–10	2–10

Printed values: *p* < 0.05, * (starred) values: *p* < 0.001.

**Table 4:** Comparison of models for sleep duration predicting mental health outcomes (bold numbers show the best model).

Outcomes	Linear Model	Quadratic Model	Fractional Polynomial Model
**Psychological distress**			
sleep duration (p)	<0.001	0.002	
sleep duration squared (p)	n/a	0.033	n/a
sleep duration (power → p)	n/a	n/a	1 → <0.001
age (power → p)			3 → <0.001
adjusted R^2^	0.130	0.133	**0.134**
RMSE	0.933	0.931	**0.931**
AIC	2745.9	2743.3	**2740.9**
**Sense of coherence**			
sleep duration (p)	<0.001	0.002	
sleep duration squared (p)	n/a	0.018	n/a
sleep duration1 (power → p)	n/a	n/a	2 → <0.001
sleep duration2 (power → p)	n/a	n/a	3 → <0.001
adjusted R^2^	0.190	0.194	**0.199**
RMSE	0.890	0.888	**0.886**
AIC	2523.7	2520.1	**2514.2**
**Life satisfaction**			
sleep duration (p)	<0.001	0.021	
sleep duration squared (p)	n/a	0.132	n/a
sleep duration (power → p)	n/a	n/a	1 → <0.001
adjusted R^2^	0.224	**0.225**	0.224
RMSE	0.871	**0.871**	0.871
AIC	2673.9	**2673.6**	2673.9
**Work ability**			
sleep duration (p)	<0.001	<0.001	
sleep duration squared (p)	n/a	<0.001	n/a
sleep duration (power → p)	n/a	n/a	−1 → <0.001
adjusted R^2^	0.075	**0.086**	0.079
RMSE	0.958	**0.952**	0.955
AIC	2903.4	**2891.5**	2898.0

**Table 5:** Estimates in the best models for mental health outcomes.

Sleep as Main Effect Variable and Covariates	Outcome Indicators of Mental Health			
	Psychological Stress Estimate (SE)	Sense of Coherence Estimate (SE)	Life Satisfaction Estimate (SE)	Work Ability Estimate (SE)
Model	Frac. polynomial	Frac. polynomial	Quadratic	Quadratic
sleep duration (hours)	n/a	n/a	0.406 (0.18) *	0.866 (0.15) ***
sleep duration squared	n/a	n/a	−0.019 (0.01)	−0.051 (0.01) ***
sleep duration power1	−0.173 (0.03) ***	0.096 (0.02) ***	n/a	n/a
sleep duration power2	n/a	−0.008 (0.002) **	n/a	n/a
gender (female vs male)	0.096 (0.06)	0.016 (0.06)	0.196 (0.06) **	0.186 (0.06) **
age (years)	n/a	0.013 (0.002) ***	0.001 (0.002)	−0.003 (0.002)
age (years) power	−0.017 (0.0004) ***	n/a	n/a	n/a
living w/partner (yes vs no)	−0.051 (0.06)	0.353 (0.06) **	0.352 (0.05) ***	0.130 (0.06)
education (tertiary vs secondary)	−0.073 (0.09)	0.303 (0.10) **	0.300 (0.08) ***	0.059 (0.10)
employment position (manager vs non-manager)	−0.200 (0.07) **	0.341 (0.08) ***	0.268 (0.06) ***	0.401 (0.07) ***
social support (optimal vs suboptimal)	−0.589 (0.07) ***	0.639 (0.06) ***	0.750 (0.06) ***	0.401 (0.06) ***

* *p* < 0.05, ** *p* < 0.01, *** *p* < 0.001.

## Data Availability

Not applicable.
